# Analysis of the 56-kDa type specific antigen gene of *Orientia tsutsugamushi* from northern Vietnam

**DOI:** 10.1371/journal.pone.0221588

**Published:** 2019-08-30

**Authors:** Nguyen Vu Trung, Le Thi Hoi, Do Duy Cuong, Doan The Ha, Tran Mai Hoa, Vu Ngoc Lien, Nguyen Thi Hoa, Le Nguyen Minh Hoa, Dang Thi Huong, Vu Thi Ngoc Bich, H. Rogier van Doorn, Behzad Nadjm, Allen L. Richards

**Affiliations:** 1 National Hospital for Tropical Diseases, Hanoi, Vietnam; 2 Hanoi Medical University, Hanoi, Vietnam; 3 Bach Mai Hospital, Hanoi, Vietnam; 4 Oxford University Clinical Research Unit, Wellcome Trust Asia Program, Hanoi, Vietnam; 5 Naval Medical Research Center, Silver Spring, Maryland, United States of America; 6 Uniformed Services University of the Health Sciences, Bethesda, Maryland, United States of America; Johns Hopkins University, UNITED STATES

## Abstract

Scrub typhus has been documented since 1932 in Vietnam, however, the disease burden of scrub typhus remains poorly understood in the country. We conducted this study to describe the phylogenetic analysis of the 56-kDa type-specific antigen (TSA) gene of *Orientia tsutsugamushi* associated with PCR positive cases of scrub typhus. Of 116 positive samples, 65 type-specific antigen gene sequences were obtained and classified into 3 genogroups: Karp, Kato and Gilliam. The Karp genogroup was the most frequently detected phylogenetic cluster in the study with 30 samples (46%), followed by Kato and Gilliam with 20 (31%) and 15 (23%), respectively. All sequences showed 94–100% nucleotide similarity to reference sequences collected in the central part of Vietnam in 2017. Patients infected with Karp genogroup were more likely to have significant thrombocytopenia than the other genogroups. These results suggest that any scrub typhus vaccine considered for use in Vietnam should provide protection against each of these 3 genogroups.

## Introduction

Rickettsioses are arthropod-borne zoonoses caused by intracellular bacteria of the genera *Rickettsia* and *Orientia* belonging to the family Rickettsiaceae. These small, Gram-negative, obligate intracellular bacteria are transmitted to humans through bites and feces of infected arthropod vectors, such as fleas, mites, ticks, and lice. The clinical diseases associated with Rickettsiaceae infections are classified into three major groups: scrub typhus group (STG), spotted fever group (SFG), and typhus group (TG).

Rickettsial infections have been documented in Vietnam since 1932 [1] and represent a major cause of febrile illnesses among residents of Southeast Asia and returning travelers, and are also a leading cause of treatable non-malarial febrile illness [2]. There are few recent reports describing the prevalence and genetics of scrub typhus in Vietnam. In Hanoi between 2001–2003, 41% and 33% of adult hospital admissions with acute undifferentiated fever (after exclusion of malaria, dengue and typhoid fever) had laboratory confirmed scrub typhus and murine typhus, respectively [3]. A subsequent study determined the seroprevalence of rickettsioses within rural and urban populations of northern Vietnam during 2011–2012. The prevalence of typhus group rickettsiae–specific antibodies was significantly greater than scrub typhus group orientiae- or spotted fever group rickettsiae–specific antibodies (P < 0.05) [4]. Le et al conducted genetic analysis of the 56-kDa type specific antigen (TSA) gene in central Vietnam in 2015 and demonstrated that the 14 sequences were related to 4 genogroups: Karp, Kawasaki, Gilliam (JG-v and TG-v) and TA716 with the majority sequences associated with the Karp genogroup (64.4%) [5]. Nguyen et al collected samples from 63 patients with typical signs and syndrome of rickettsial disease and determined 42 positive for scrub typhus during 2015–2016 in northern Vietnam. Using 56-kDa TSA gene sequencing they found that the most common genotype identified to be Karp (55%), following by TA763 (17%), Gilliam type in Japan variant (17%), and Kato (12%) [6].

The antigenic variation of *O*. *tsutsugamushi* depends largely on diversity in the immune-dominant 56-kDa TSA located on the surface of the bacterial membrane [7],[8]. According to this antigenic variation, there were initially three major serotypes of *O*. *tsutsugamushi* described: Gilliam, Karp, and Kato [9]. Many other serotypes have subsequently been described since, such as Kawasaki, Kuroki, Boryoung, and Shimogoshi [9]. Groves and Osterman (1978) showed that the virulence between serotypes of *O*. *tsutsugamushi* was associated with genetic differences between mouse strains [8].

Genetic typing of orientiae, for the most part has been attributed to the genetic variation of the TSA gene [10]. To date, there have been few reports of phylogenetic analysis of the 56-kDa TSA genes of *O*. *tsutsugamushi* isolates obtained from patients with scrub typhus, and corresponding differences in clinical features between the genotypes in northern Vietnam. The lack of knowledge of the variability of the 56-kDa TSA genes and their products among orientiae in northern Vietnam could have an important effect on the accuracy of diagnostic tests used, and in vaccine development for and epidemic disease control of scrub typhus in endemic countries like Vietnam [11].

Diagnosis of scrub typhus is notoriously difficult for a number of reasons: 1) clinical presentations are non-specific and highly variable; 2) the organisms do not grow on routine culture media, and require cumbersome biosafety level 3 procedures; and 3) despite significant advances in diagnostic methodology in recent years, there remains a general lack of standardized and validated assays, and lack of consistent practice across different laboratories [12]. A recent study reporting the use of eschar swabbing for molecular diagnosis and genotyping of *O*. *tsutsugamushi* appears very useful for the rapid detection in the early phase of infection [5].

We conducted a prospective observational study of clinically suspected rickettsial fever cases in the National Hospital for Tropical Diseases (NHTD) and Bach Mai hospital, two tertiary care hospitals in Hanoi, Vietnam, from 2015 to 2017. In this report, we described the clinical characteristics of scrub typhus cases confirmed by species-specific qPCR assays and linked the disease presentations with the phylogenetic analysis of the 56kDa TSA gene of *O*. *tsutsugamushi*.

## Materials and methods

### Patient data and study design

We conducted a prospective observational hospital-based study of clinically suspected rickettsiosis in the National Hospital for Tropical Diseases and Bach Mai Hospital, two large referral hospitals in Hanoi, Vietnam, from March 2015 to December 2017. Patients were included in this study if they fulfilled all three following criteria:1) age over 15 years; 2) fever (> 37.5°C) of unknown cause or presence of an eschar; and 3) had at least one of the following clinical signs or symptoms: rash, headache, myalgia, lymphadenopathy, hepatomegaly, or splenomegaly.

Information including demographics, medical history, clinical and laboratory findings, and treatment provided were collected into a case record form. Outcome was recorded at discharge, and classified as full recovery, death, or palliative care discharge. Palliative care discharge is a commonly preferred alternative to dying in the hospital in Vietnam, where a patient for whom ongoing care is considered futile is discharged to permit them to die in their home with their family.

For evaluation of disease severity, the APACHE II (Acute Physiology and Chronic Health Evaluation II) scores were determined upon admission. Patients with an APACHE II score ≥ 10, measured within 24 hours of admission, were regarded as severe cases (in other settings this has corresponded to a hospital case fatality rate of over 10% in non-surgical patients [13]).

#### PBMC isolation

For each enrolled patient, five milliliters of whole blood were collected into EDTA tubes. Blood was then added to Ficoll gradient (Sigma-Aldrich, Germany) for isolation of lymphocytes from human peripheral blood and centrifuged at 1,800 G for 10 minutes. The plasma layer was removed and PBMCs were collected into new tubes. PBMCs were subsequently stored at -80°C prior to nucleic acid extraction.

#### Eschar biopsy collection

The eschars to be biopsied were punched out using disposable, sterile punches of varying sizes. The size of the biopsies ranged from 3–10 mm.

#### DNA extraction

Clinical samples were subsequently manually processed using DNA extraction kits (Qiagen, Hilden, Germany) according to the manufacturer’s instructions. For each patient, 200μl of PBMCs were used for DNA extraction. Each eschar biopsy specimen was immersed in lysis buffer for 1 hour and 200μl of the supernatant was subsequently collected for DNA extraction. Extracted DNA from each specimen was eluted into 100μl of Tris EDTA (TE) buffer for molecular analysis. After extraction, sample DNA was stored at -80°C.

#### *Orientia tsutsugamushi*-specific qPCR assay

DNA preparations from clinical samples were tested using the Otsu47 quantitative real-time polymerase chain reaction (qPCR) assay with *O*. *tsutsugamushi—*specific primers and probes as described previously [14].

#### Sequencing of the 56 kDa type specific antigen gene

Samples positive for *O*. *tsutsugamushi* were submitted to conventional PCR amplification prior to sequencing. We developed in-house primers targeting a 400 bp fragment of the *O*. *tsutsugamushi* 56 kDa TSA gene which also includes the variable regions (Table 1).

**Table 1:** Primers for conventional PCR amplification prior to sequencing.

Otr56-573F (OtsuF)	AATTGCTAGTGCAATGTCTG
Otr56-980R (OtsuR)	GGCATTATAGTAGGCTGAG
Otr56-498F	AATTAGTTTAGAATGGTTACCAC
Otr56-1459R	TCTGTATCTGTTCGACAGATGCACTATTAG

Amplification products were separated by electrophoresis on a 1.5% agarose-tris-borate-EDTA gel. PCR products were sequenced using the BigDye Terminator kit (Applied BioSystems, USA) on an ABI PRISM 3130 Genetic Analyzer (Applied BioSystems). The sequences were analyzed using the ABI PRISM DNA Sequencing Analysis software version 3.0 (Applied BioSystems) and compared to sequences available in GenBank using the BLAST algorithm (http://blast.ncbi.nlm.nih.gov/Blast.cgi). Sample sequences are available in GenBank with accession numbers MG735127 to MG735183, MG872767 to MG872771 and MG920487 to MG920489 (S2 Table).

Phylogenetic analysis of *O*. *tsutsugamushi* sequences of the 56-kDa TSA gene was conducted to determine the genetic relatedness with previous isolates from Vietnam [5],[6],[15]. A total 65 fragments of 56-kDa gene nucleotide sequences of the variable region were obtained and submitted to GenBank. These sequences were aligned using CLUSTALW and phylogenetic inferences were obtained using MEGA 7.0.26 software using the Neighbor-Joining method. Genetic distances were calculated using the Kimura two-parameter distance algorithm. Bootstrap support values above 70 were considered significant with bootstrap performed 1,000 times.

#### Statistical analysis

Median and interquartile ranges were used for the description of non-normally distributed continuous variables. Categorical variables were summarized as frequencies and percentages. The Fisher’s exact test was used for the comparison of clinical characteristics, laboratory results and treatment outcome among groups. Non-normally distributed data were compared by using the Kruskal-Wallis test for the three-group comparison. These analyses were performed in STATA 12.0.

#### Ethical considerations

All enrolled patients provided written informed consent. For patients between 15–17 years old, consent was obtained from both patient and their relatives. The study protocol was approved by the Institutional Review Boards of the National Hospital for Tropical Diseases and Bach Mai Hospital.

## Results

### Patient demographic and clinical information

Among 402 scrub typhus suspected cases enrolled between March 2015 and December 2017, 116 were positive for *O*. *tsutsugamushi* by qPCR from either blood or eschar biopsy samples. From these 116 qPCR positive individuals, 63 patients yielded 65 sequences of the 56kDa TSA gene and 54 individuals’ PBMC samples did not produce amplicons for sequencing after multiple PCRs. Two scrub typhus patients provided both PBMC and eschar samples for which sequences were obtained. The other 61 patients provided only PBMC samples and therefore only had single sequences. One unique patient who had two different *O*. *tsutsugamushi* genogroups sequenced (one from their eschar and one from their PBMC samples) was excluded from further analysis.

Among the 62 patients for whom genetic analysis were performed, 28 (45%) were male with a median age of 53 (39–63); 43 (69%) and 16 (26%) participants lived in rural areas outside of Hanoi province and in Hanoi province, respectively; 36 (58%) were farmers; and 46 (74%) were treated with antibiotics in another hospital before their transfer to the study hospitals.

The most common clinical features among the 62 confirmed scrub typhus patients, other than fever, were hyperaemic skin 56 (90%), headache 53 (86%), myalgia 45 (73%) and conjunctivitis 54 (87%). Rash (other than hyperaemia) was seen in 26 (42%) of patients, lymphadenopathy in 17 (27%) and hepatomegaly in 5 (8%). Eschars were found in 40 (65%) of cases with scrub typhus and the median duration of hospitalization was 7 (5–10) days (S1 Table).

**Fig 1:**
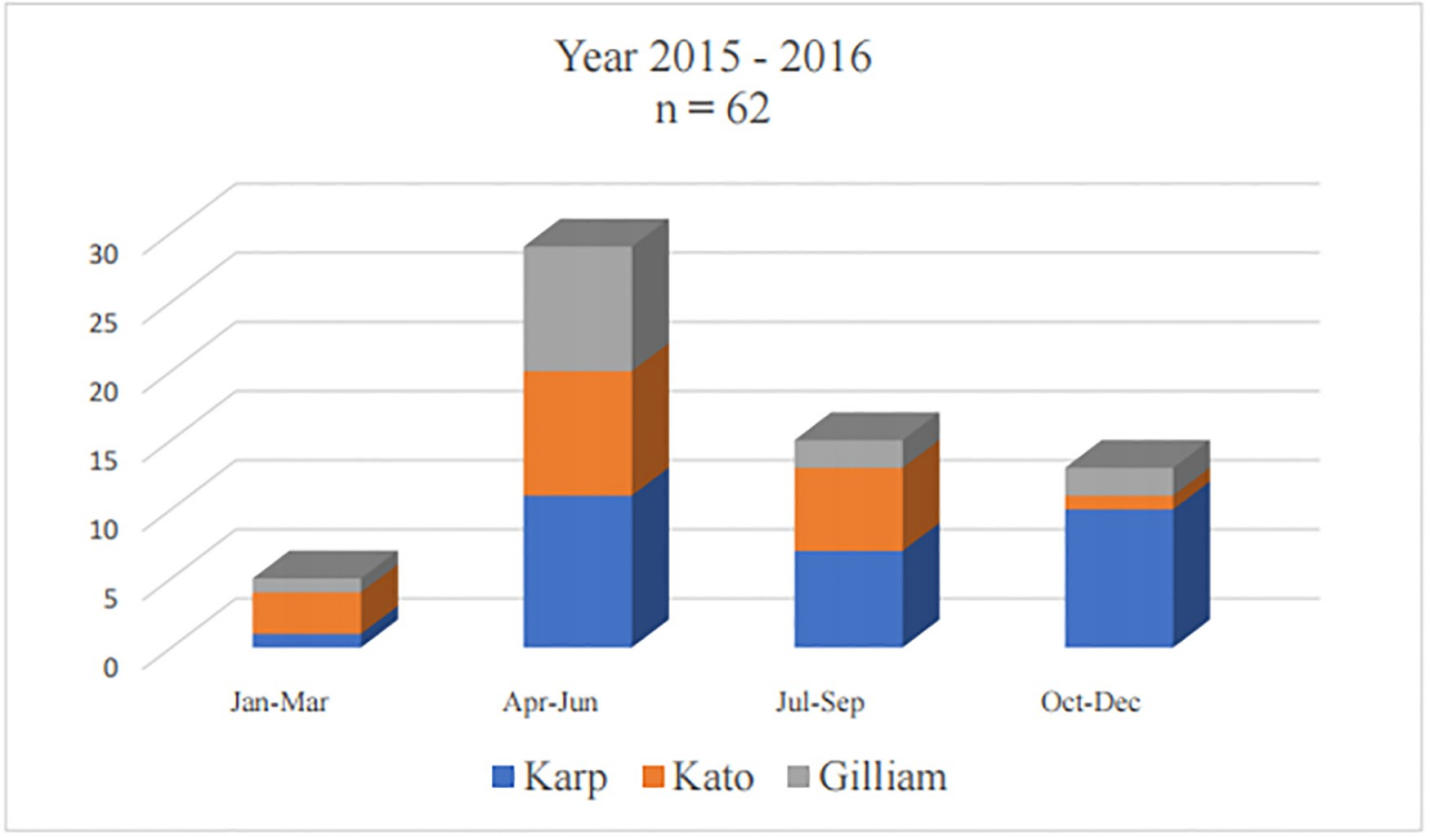
Month of hospital presentation of circulating genogroups during 2015–2016.

The Karp genogroup accounted for the highest proportion 46% (30/65), followed by the Kato and Gilliam genogroups with 31% (20/65) and 23% (15/65), respectively. All genogroups were found circulating throughout the year. However, Karp genogroup was the dominant group found during October-December, whilst Kato genogroup was dominant identified in January-March. During the peak season (the wet/summer months of April-June), all three genogroups were well represented (Fig 1). In 2017, only 3 cases (2 from April-June and 1 from July-September) were recruited in this study and therefore was not added to Fig 1.

### Associations between genogroups and disease severity

We analyzed the associations between the three genogroups and clinical presentations and ascertained that there was no significant association between genogroup and patient outcome. The Karp genogroup showed the highest proportion of patients with an eschar and lowest proportion of rash compared to other genogroups, though this was not statistically significant (S1 Table). The highest proportion of patients with APACHE II score > = 10 occurred in the Karp genogroup, though this was not statistically significant (p = 0.541). Among other factors assessed including demographics, clinical features and outcomes, only the platelet count was significantly different among the three genogroups with a p-value = 0.009 (S1 Table). The Karp genogroup had a lower platelet count in comparison with the others two groups (S1 Table).

### Genotype classification and phylogenetic analysis of *O*. *tsutsugamushi* from northern Vietnam

**Fig 2:**
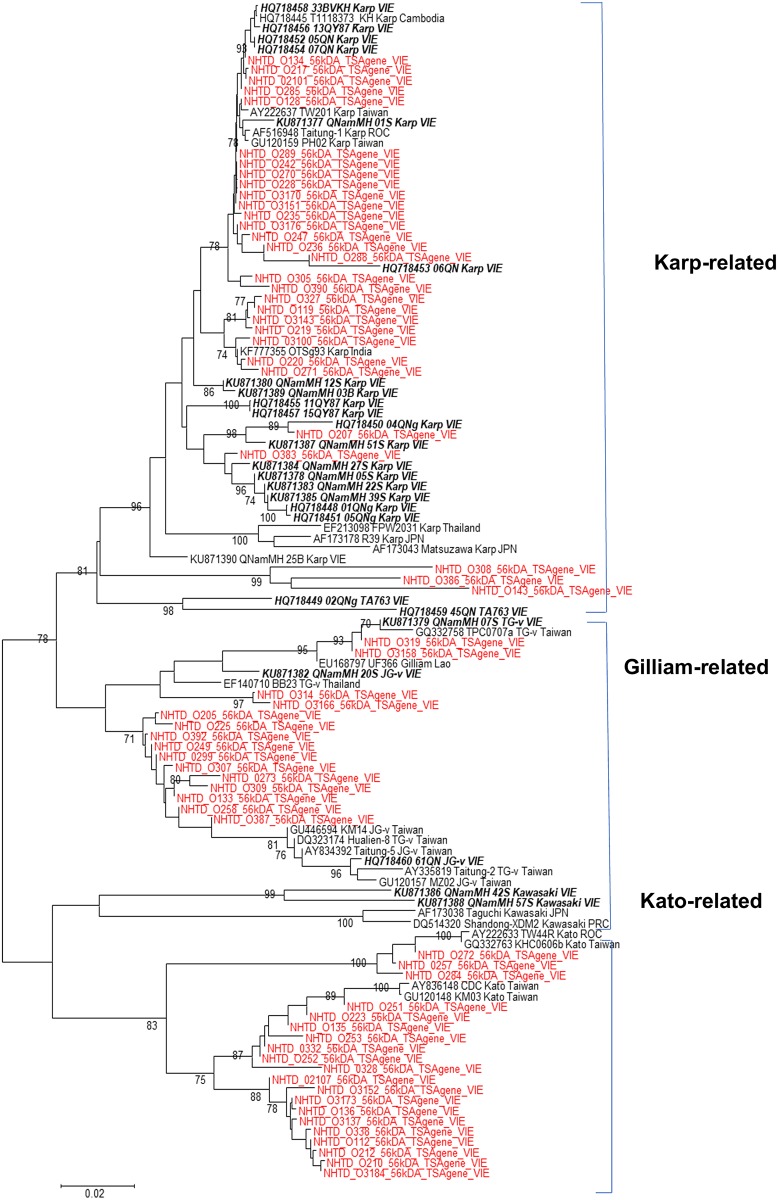
Phylogenetic tree of *O*. *tsutsugamushi*, based on 56-kDa TSA gene sequences. Sequences from this study are indicated with red font and other sequences from Vietnam in bold italic black font. Sequences from other countries are indicated with regular black font.

All sequences from our research formed three major branches related to the three genogroups: Karp, Kato and Gilliam (Fig 2). The most frequent genotypes determined were in the Karp genogroup, with 95–100% nucleotide identity to the reference genomes (accession number HQ718453 and KU871377 from central Vietnam). The Kato genogroup had the second highest number of sequences with a percentage nucleotide identity ranging from 96% to 100% when compared to Kato references (strains AY836148 and GQ332763 from Taiwan). The remaining sequences belonged to the Gilliam genogroup with 94% to 96% nucleotide identity to the reference strains (KU871382 from Vietnam and EF140710 from Thailand). In this study, none of the sample sequences clustered with the Kawasaki, Shimokoshi, Boyong, Kuroki, TA678, or TA716 genogroups.

**Fig 3:**
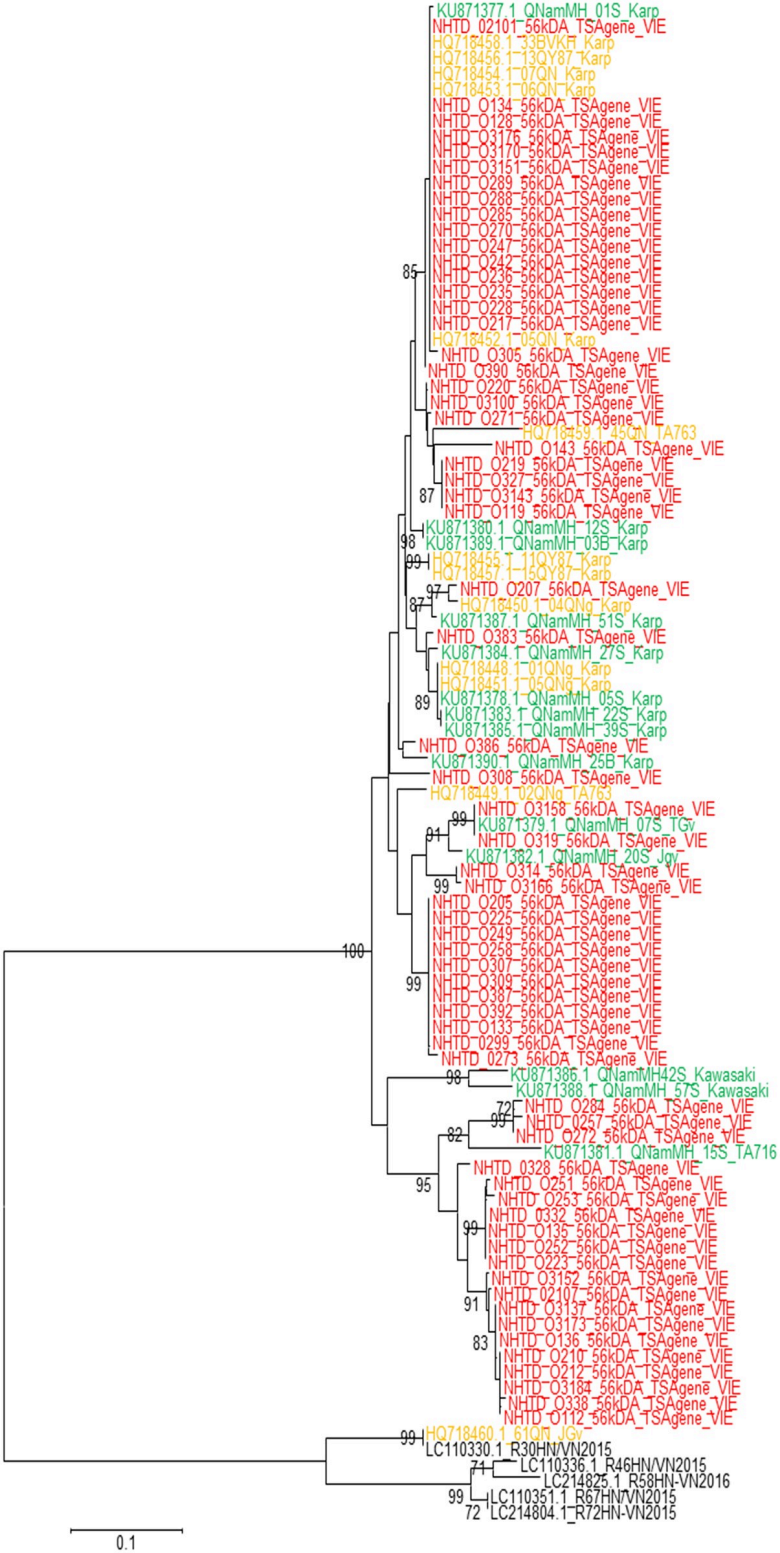
Phylogenetic tree of 56kDa TSA gene *Orientia tsutsugamushi* sequences detected in Vietnam. This subset consists of sequences from this study (red font), 14 sequences from the reference [5] (green font), 13 sequences from the reference [15] (orange font) from central Vietnam, and 5 sequences from northern Vietnam, the reference [6] (black font).

We did not study the similarity between sequences in our study and the study by Nguyen et al [6] because the target genes for sequencing in these two studies were different. There was a close relationship among sequences in our study and sequences from central Vietnam [5] with percentage nucleotide identity ranging from 94 to 100% as mentioned above (Fig 3).

We compared genogroups from different sample types (blood and eschar). Two patients were tested with both blood and eschar biopsy samples. The results showed that the same genotype of the Kato genogroup was detected in both samples in the first patient and in the second patient, a genotype of the Karp genogroup was detected in blood and a separate genotype of the Gilliam genogroup was detected in eschar biopsy (S2 Table).

For all three genogroups identified, the sequences were mostly found in Hanoi and surrounding provinces. The three genogroups were also well distributed through most parts of northern Vietnam except for the highland areas which could not be investigated due to their remoteness.

**Fig 4:**
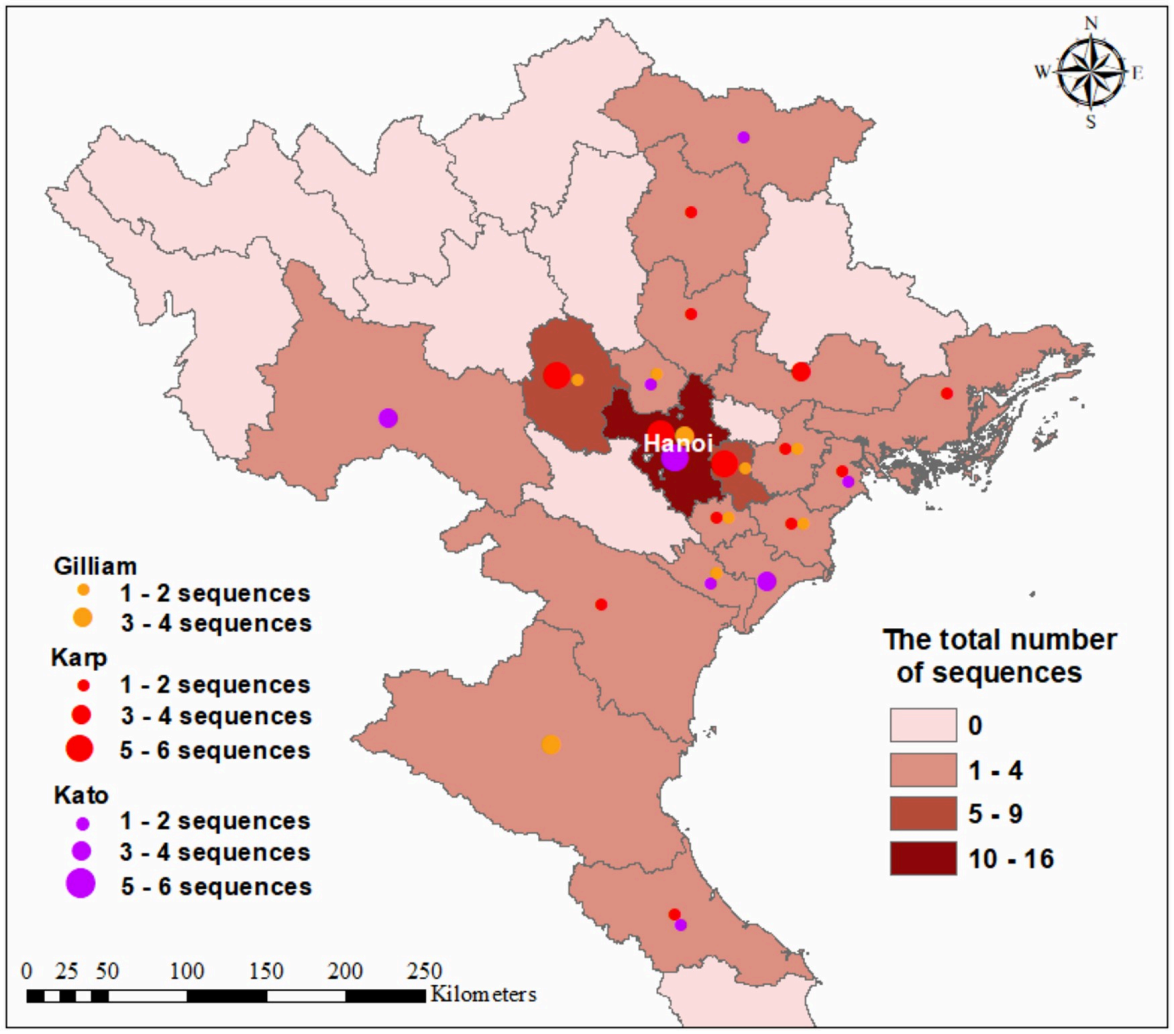
Mapping of *Orientia tsutsugamushi* genogroups distribution in northern Vietnam. The map was generated using software ArcGIS 10.1 based on the geospatial data from the Center for Spatial Sciences at the University of California, Davis.

The number of samples for which genogroups were identified were distributed similarly/dissimilarly with qPCR positive patients (Fig 4).

## Discussion

### The prevalence of *O*. *tsutsugamushi* genogroups in this study

Genotyping based upon the 56 kDa TSA gene sequences (400 bp fragment) obtained from clinical specimens of scrub typhus patients of northern Vietnam showed “some” similarity and “some” dissimilarity among the three genogroups Karp, Kato and Gilliam. Notably, genotypes that were not encounter include those of the genogroups Kawasaki, Shimokoshi, Boyong, Kuroki, TA678, TA 763, or TA716 which have been reported from Japan [16], Thailand [7],[17], and Cambodia [15].

Among the Karp, Gilliam and Kato genogroups found in our study, the Karp genogroup was most frequently detected (46%). In an earlier study in Vietnam, the Karp strain was also found to predominated (77%), and TA763 (15.5%) and JG-v (7.5%) strains less frequently detected [15]. There were four genogroups described in Quang Nam province including Karp, Kawasaki, Gilliam (JG-v and TG-v) and TA716 and the majority (9/14; 64.4%) of contemporary *O*. *tsutsugamushi* genogroups were related to the Karp genogroup [5]. According to the articles by Duong et al and Blacksell et al, the distribution of genogroups among clinical isolates from Cambodia and Vietnam, Karp and Karp-like genogroups were also dominant [17]. However, they also found other groups such as TA763 and JG-v circulating in the central part of Vietnam during 2008–2010 (Table 2) [15]. In Nguyen’s study, 42 clinical samples sequenced found that the genogroup Karp was most common (23/42), followed by TA763 (7/42) and JGv (7/42), and lastly, Kato (5/42). Interestingly, we did not detect TA763 in our sampling nor did Le et al [5].

**Table 2:** Prevalence of current circulating genotypes of *O*. *tsutsugamushi* in Vietnam.

Study	Location	Karp	Gilliam	Kato	TA716	TA763
Duong et al (2013) n (%) [15]	Central Vietnam	10 (77)	1 (7.5)	0 (0)	0 (0)	2 (15.5)
Le et al (2016) n (%) [5]	Central Vietnam	9 (64.4)	2 (14.3)	0 (0)	1 (7.1)	0 (0)
Nguyen et al (2017) n (%) [6]	Northern Vietnam	23 (55)	7 (17)	5 (12)	0 (0)	7 (17)
This study n (%)	Northern Vietnam	30 (46)	15 (23)	20 (31)	0 (0)	0 (0)

In Japanese rodents, the diversity of the immunodominant 56-kDa TSA detected by PCR assay, also consisted of the three serotypes of *O*. *tsutsugamushi*: Gilliam, Karp, and Kato similar to our study [9]. JG-v and Kato group were also prevalent in Cambodia with proportions of 25% and 22%, respectively. As in Thailand, JG-v was the second most frequently detected strain in Central Vietnam and Cambodia with sequencing technology [15].

### The relation of genotypes and some epidemiological features

In this study, we found predominance of middle-aged or older scrub typhus patients. In terms of gender distribution, many urban females were recruited in this study and this was in line with earlier reports. In a study by Park et al, conducted on scrub typhus patients > 16 years of age with eschars in South Korea in 2006, the median age of the patients was 63 years (interquartile range, IQR, 49–71) and 41.2% were males [18]. According to a study by Le et al, performed from March to September 2015 in central Vietnam on scrub typhus patients, the mean age was 42.5 years old and 37.5% of total patients were male [5].

Sixty-five samples collected were from patients from 16 provinces/cities of Vietnam with the highest number of patients (n = 17) from Hanoi. This likely relates to the high population in Hanoi and the location of the study hospitals in Hanoi. Hanoi is the capital and largest city in northern Vietnam where the national hospital is located and several genogroups co-existed in this region. Cases in this study were mostly admitted to the hospital during April to December. *O*. *tsutsugamushi* from the Gilliam genogroup circulated sporadically through the year, while those of the Kato genogroup concentrated from January to September; and those of the Karp genogroup appeared mostly from April to December. Another study conducted in northern Vietnam (Nguyen et al) found the majority of positive samples using qPCR during July 2015 to January 2016 and from May to September 2016, and no positive cases were recorded between February and April 2016 [6].

### The relationship between genogroup and clinical characteristics and severity

Despite the lack of any significant difference in case fatality between patient infected with *O*. *tsutsugamushi* from the 3 different genogroups, there were some indications that there may be a difference in severity associated with the different genogroups. The platelet count differed significantly among three genogroups, with levels in the Karp group lower than in the two other genogroups. Thrombocytopenia has been identified as a risk factor for the development of ARDS, which is a rarely reported but serious and potentially fatal complication of scrub typhus [19],[20]. Wei et al also found that Karp groups had significant lower number of platelet count than Kato and Gilliam groups [8]. Severe manifestations in scrub typhus patients may also include pneumonitis, meningitis, encephalitis, disseminated intravascular coagulation, and multiorgan failure. Case fatality rates can go up to 50% if not treated appropriately [21].

We also observed other factors suggesting more severe manifestations in patients infected with *O*. *tsutsugamushi* of the Karp genogroup compared to *O*. *tsutsugamushi* of the Kato and Gilliam genogroups. It was observed that a greater proportion of patients infected with Karp genogroup had low albumin compared to those infected with Gilliam and Kato genogroups (p = 0.067). In addition, there was a corresponding trend towards higher rates of edema (p = 0.071) and a trend towards a higher proportion of patients with increased AST (p = 0.089) among patients infected with Karp genogroup compared to Gilliam genogroup (S1 Table). This is in keeping with data (from an animal model of *O*. *tsutsugamushi*, where three groups were designated according to virulence in mice: a highly virulent group including the Karp, Kato, KN-3 serotypes; a low virulence group including the Kuroki, Kawasaki, and KN-2 serotypes; and an intermediate virulence group including the Gilliam serotype [22]. Others have suggested that Gilliam, Karp, and Kato are all virulent strains that they can cause severe and potentially life-threatening disease in humans [18]. These studies were consistent with our finding that Karp genogroup tends to be related to more severe cases. Interestingly, we found some factors that were absent in our Kato genogroup (n = 20) including hepatomegaly, splenomegaly and hyperbilirubinemia, however these differences were not statistically significant. However, to have stronger evidence about differences in severity associated with different *O*. *tsutsugamushi* genogroups, more data linking genogroup with clinical data is needed from a variety of settings.

## Limitations

One of the limitations of the study was that this study was conducted only in Hanoi–northern Vietnam, so the *O*. *tsutsugamushi* genogroups analyzed here may not be representative of all Vietnam. Besides that, data were collected in two central hospitals, limiting recruitment of milder cases as these may be treated as outpatients or in district / provincial level hospitals and therefore they would never reach the two central hospitals. This could be a critical limitation in the elucidation of the relationship between genotype and differences in severity, and research in this field should in future involve health centers that represent locations where patients first present with scrub. In addition, a limitation was that only a 400 bp target for sequencing of the 56kDa TSA gene, including a variable portion, was used. Moreover, the success of the sequencing was found only in 63 out of 115 (54.8%) individuals’ 47 kDa qPCR positive samples (63 PBMC and 2 eschar samples), indicating that 54 patients’ samples (all PMBC derived) were not PCR amplified for sequencing. This could possibly be due to the lack of sufficient DNA or inhibitors in the samples, and/or the 56 kDa primers developed for the in-house assay did not recognize their targets because of sequence variation. Thus, many unique sequences could have been missed due to the lack of sensitivity of the developed 56 kDa PCR assay. We did not perform whole genome sequencing because of absence of laboratory capacity to culture *O*. *tsutsugamushi*.

## Conclusions

Knowledge of the genetic variability of *O*. *tsutsugamushi* obtained in this study will enhance the development and application of diagnostic methods and vaccine research. Our results also suggest that there may be differences in clinical features and severity relating to the infecting genotype. The Karp genogroup is the most frequently isolated and there is some suggestion that it causes more severe cases.

## Supporting information

S1 Table Comparison for the features of demographics, symptoms, signs, treatment, and outcomes among three genogroups. (PDF)

S2 Table Metadata of patients recruited in this study. (PDF)

## Abbreviations

bp: base pair
*O. tsutsugamushi*: *Orientia tsutsugamushi*
TSA: type-specific antigen
