# Supplementary material for: Analysis of the 56-kDa type specific antigen gene of Orientia tsutsugamushi from northern Vietnam
Source: PLoS One. 2019 Aug 30;14(8):e0221588. doi: 10.1371/journal.pone.0221588 (PMC6716651; doi:10.1371/journal.pone.0221588)
Supplement: S1 Table — (PDF) [file pone.0221588.s001.pdf]

**Characteristics**	**Gilliam** (n = 14)	**Karp** (n = 29)	**Kato** (n = 19)	**Total** (n = 62)	**p-value 3 groups**	**p-value Karp vs Kato**	**p-value Gilliam vs Karp**
**Demographics**							
Age (median (p25- p75))	60<br>36 - 75	52<br>39 – 60	51<br>37 - 63	53<br>39 - 63	0.934[1]	0.598[1]	0.358[1]
Male (n (%))	7 (50.0)	15 (51.7)	6 (31.6)	28 (45.2)	0.408[2]	0.237[2]	1.0[2]
Living in Hanoi (n (%))	4 (28.6)	6 (20.7)	6 (31.6)	16 (25.8)	0.698[2]	0.501[2]	0.704[2]
Living in rural location (n (%))	9 (64.3)	19 (65.5)	15 (79.0)	43 (69.4)	0.597[2]	0.354[2]	1.0[2]
Admitted in Summer (n (%))	9 (64.3)	24 (82.8)	15 (79.0)	48 (77.4)	0.451[2]	1.0[2]	0.252[2]
Farmer (n (%))	8 (57.1)	19 (65.5)	9 (47.4)	36 (58.1)	0.453[2]	0.245[2]	0.739[2]
**Symptoms on admission**							
Headache (n (%))	13 (92.9)	25 (86.2)	15 (79.0)	53 (85.5)	0.653[2]	0.695[2]	1.0[2]
Myalgia (n (%)) (n = 48)	11 (78.6)	21 (72.4)	13 (68.4)	45 (72.6)	0.932[2]	1.0[2]	1.0[2]
Retro-orbital-pain (n (%))	3 (21.4)	7 (24.1)	3 (15.8)	13 (21.0)	0.953[2]	0.749[2]	1.0[2]
Sorethroat (n (%))	4 (28.6)	5 (17.2)	2 (10.5)	11 (17.7)	0.261[2]	0.596[2]	0.445[2]
Cough (n (%))	5 (35.7)	14 (48.3)	8 (42.1)	27 (43.6)	0.574[2]	0.771[2]	0.369[2]

Nausea (n (%))	3 (21.4)	11 (37.9)	8 (42.1)	22 (35.5)	0.426[2]	0.466[2]	0.574[2]
Vomiting (n (%))	2 (14.3)	9 (31.0)	5 (26.3)	16 (25.8)	0.412[2]	0.391[2]	0.555[2]
Abdominal pain (n (%))	0 (0)	6 (20.7)	1 (5.3)	7 (11.3)	0.095[2]	0.182[2]	0.117[2]
Diarrhea (n (%))	2 (14.3)	9 (31.0)	1 (5.3)	12 (19.4)	0.061[2]	**0.025[2]**	0.474[2]
**Physical signs at admission**							
Congested skin (n (%))	13 (86.7)	26 (89.7)	17 (85.0)	56 (90.3)	0.893[2]	0.669[2]	1.0[2]
Conjunctivitis (n (%))	12 (85.7)	26 (89.7)	16 (84.2)	54 (87.1)	0.932[2]	1.0[2]	1.0[2]
Eschar (n (%))	8 (57.1)	21 (72.4)	11 (57.9)	40 (64.5)	0.554[2]	0.357[2]	0.488[2]
Rash (n (%))	6 (42.9)	11 (37.9)	9 (47.4)	26 (41.9)	0.906[2]	0.780[2]	0.768[2]
Lymphadenopathy (n (%))	6 (42.9)	9 (31.0)	2 (10.5)	17 (27.4)	0.208[2]	0.238[2]	0.652[2]
Hepatomegaly (n (%))	1 (7.1)	4 (13.8)	0 (0)	5 (8.1)	0.233[2]	0.405[2]	1.0[2]
Splenomegaly (n (%))	1 (7.1)	3 (10.3)	0 (0.0)	4 (6.5)	0.433[2]	0.703[2]	1.0[2]
Edema (n (%))	1 (7.1)	10 (34.5)	3 (15.8)	14 (22.6)	0.110[2]	0.197[2]	0.071[2]
Rales (n (%))	3 (21.4)	11 (37.9)	4 (21.1)	18 (29.0)	0.422[2]	0.341[2]	0.324[2]
Decreased breath sounds (n (%))	1 (7.1)	4 (13.8)	4 (21.1)	9 (14.5)	0.653[2]	0.695[2]	1.0[2]
Convulsion(n (%))	1 (7.1)	4 (13.8)	3 (15.8)	8 (12.9)	0.891[2]	1.0[2]	1.0[2]
**Laboratory test at admission**							

Erythrocyte (T/l) (median (IQR))	4.15<br>(3.17 - 4.45)	4.15<br>(3.84 - 4.8)	4.63<br>(4.01 – 4.87)	4.23<br>(3.84 – 4.8)	0.263[1]	0.352[1]	0.422[1]
Leukocyte (G/l) (median (IQR))	10.7<br>(7.8 -11.2)	9.1<br>(5.7 – 11.1)	7.8<br>(6.7 – 11.4)	9.1<br>(6.6 – 11.2)	0.396[1]	0.638[1]	0.223[1]
Platelet (G/l) (median (IQR))	167<br>(95 - 220)	69<br>(48 - 95)	125<br>(84 - 169)	95<br>(62 - 169)	**0.009[1]**	**0.043[1]**	**0.007[1]**
ALT >60 IU/L (n (%))	12 (85.7)	24 (82.8)	13 (72.2)	49 (80.3)	0.645[2]	0.473[2]	1.0[2]
AST >60 IU/L (n (%))	9 (64.3)	26 (89.7)	14 (77.8)	49 (80.3)	0.134[2]	0.403[2]	0.089[2]
Bilirubin Total>60µmol/l (n (%))	0 (0)	3 (18.8)	0 (0)	3 (7.9)	0.171[2]	0.262[2]	0.238[2]
Albumin < 32 g/l (n (%))	4 (40.0)	16 (80.0)	6 (50.0)	26 (61.9)	0.067[2]	0.119[2]	**0.045[2]**
Creatinine >120 µmol/ln(n (%))	1 (7.1)	7 (24.1)	2 (11.1)	10 (16.4)	0.402[2]	0.449[2]	0.240[2]
PCT >0.25 ng/ml (n (%))	8 (66.7)	24 (85.7)	11 (78.6)	43 (79.6)	0.408[2]	0.668[2]	0.211[2]
CRP > 12 mg/l (n (%))	13 (92.9)	20 (80.0)	15 (100.0)	48 (88.9)	0.119[2]	0.137[2]	0.391[2]
**Outcomes**							
Death and Palliative discharge<br>(n (%))	0 (0)	2 (6.9)	2 (10.5)	4 (6.5)	0.806[2]	1.0[2]	1.0[2]

Days to defervescence (median (IQR))	3<br>(3 - 5)	3<br>(2 - 5)	4<br>(2 - 6)	3<br>(2 – 5)	0.879[1]	0.848[1]	0.588[1]
Length of hospital stay (days)<br>(median (IQR))	6<br>(4 - 9)	7<br>(5 - 10)	7.5<br>(6 - 10)	7<br>(5 - 10)	0.414[1]	0.743[1]	0.282[1]
**Severity**							
APACHE II score ≥ 10	3<br>(21.4)	11<br>(37.9)	6<br>(31.2)	20<br>(32.3)	0.541[2]	0.763[2]	0.321[2]

[1]Mann-Whitney U test

[2] Fisher's exact test
