# Supplementary material for: Analysis of the 56-kDa type specific antigen gene of Orientia tsutsugamushi from northern Vietnam
Source: PLoS One. 2019 Aug 30;14(8):e0221588. doi: 10.1371/journal.pone.0221588 (PMC6716651; doi:10.1371/journal.pone.0221588)
Supplement: S2 Table — (PDF) [file pone.0221588.s002.pdf]

PATIENT ID	PCR CODE	AGE	SEX	LOCATION	ADMISSION	GROUP	ACCESSIOM NUMBER	PERCENTAGE NUCLEOTIDE IDENTITY	GENE REFERENCES
1	5	33	F	Ninh Binh	23/4/15	Gilliam	MG735143	95%	EF140710
2	25	75	F	Ha Noi	8/9/2015	Gilliam	MG735148	94%	EF140710
3	49	76	M	Nghe An	6/2/2016	Gilliam	MG735153	95%	EF140710
4	58	81	F	Ha Noi	11/4/2016	Gilliam	MG735154	96%	EF140710
5	7	53	M	Nghe Am	21/4/15	Gilliam	MG735160	95%	EF140710
6	14	36	M	Hai Duong	26/5/15	Gilliam	MG735163	94%	EF140710
7	19	28	M	Ha Nam	10/6/2015	Gilliam	MG735164	96%	KU871382
8	87	63	F	Hung Yen	21/10/15	Gilliam	MG735168	95%	EF140710
9	92	35	F	Quang Ninh	29/11/15	Gilliam	MG735170	95%	EF140710
10	158	50	F	Phu Tho	2/6/2016	Gilliam	MG735173	96%	KU871382
11	166	58	M	Ha Noi	12/6/2016	Gilliam	MG735174	94%	EF140710
12	33	81	F	Thai Binh	26/7/2016	Gilliam	MG735179	96%	EF140710
13	9	32	F	Hung Yen	7/5/2015	Gilliam	MG735162	96%	EF140710
14	73	32	M	Ha Noi	17/6/2016	Gilliam	MG872768	95%	EF140710
15	99	69	M	Nghe An	11/6/2017	Gilliam	MG920487	96%	EF140710
16	7	50	F	Thai Binh	2/5/2015	Karp	MG735144	1005	KU871377
17	17	74	F	Bac Giang	7/6/2015	Karp	MG735145	99%	KU871377
18	19	37	F	Thai Nguyen	1/7/2015	Karp	MG735146	98%	HQ718453
19	20	48	M	Bac Kan	1/7/2015	Karp	MG735147	98%	HQ718453
20	28	58	M	Quang Ninh	9/10/2015	Karp	MG735182	98%	HQ718453
21	35	67	F	Hung Yen	17/11/15	Karp	MG735149	99%	HQ718453
22	36	67	M	Ha Tinh	17/11/15	Karp	MG735150	100%	HQ718453
23	42	54	F	Hai Duong	13/8/2015	Karp	MG735151	100%	HQ718453

24	47	88	M	Thanh Hoa	4/2/2016	Karp	MG735152	99%	HQ718453
25	70	49	F	Ha Noi	8/6/2016	Karp	MG735155	100%	HQ718453
26	71	20	F	Thanh Hoa	15/6/2016	Karp	MG735156	98%	HQ718453
27	85	58	F	Ha Noi	3/10/2016	Karp	MG735157	99%	KU871377
28	88	52	M	Ha Noi	26/10/2016	Karp	MG735158	100%	HQ718453
29	89	76	M	Ha Noi	29/10/2016	Karp	MG735159	100%	HQ718453
30	5	67	F	Phu Tho	18/4/15	Karp	MG735183	99%	HQ718453
31	8	32	F	Hung Yen	7/5/2015	Karp	MG735161	95%	HQ718453
32	27	42	M	Quang Ninh	26/6/15	Karp	MG735165	97%	HQ718453
33	83	39	M	Phu Tho	15/10/15	Karp	MG735166	97%	KU871377
34	86	62	F	Ha Nam	20/10/15	Karp	MG735167	97%	KU871377
25	90	58	F	Ha Noi	26/11/15	Karp	MG735169	99%	HQ718453
36	143	39	M	Hai Phong	17/5/2016	Karp	MG735171	98%	KU871377
37	151	42	M	Bac Giang	29/5/2016	Karp	MG735172	100%	HQ718453
38	170	46	M	Hung Yen	22/6/2016	Karp	MG735175	99%	HQ718453
39	176	54	M	Hung Yen	1/7/2016	Karp	MG735176	100%	HQ718453
40	19	63	F	Bac Giang	28/05/2016	Karp	MG735177	98%	HQ718453
41	28	76	F	Ha Noi	6/7/2016	Karp	MG735178	99%	KU871377
42	34	60	F	Phu Tho	18/8/2016	Karp	MG735180	99%	KU871377
43	43	79	M	Phu Tho	13/10/2016	Karp	MG735181	95%	HQ718453
44	100	36	M	Ha Noi	13/8/2015	Karp	MG872771	98%	HQ718453
45	101	53	F	Ha Tinh	15/6/2017	Karp	MG920488	99%	KU871377
46	23	58	F	Son La	27/8/15	Kato	MG735129	100%	AY836148
47	51	36	M	Ha Noi	7/3/2016	Kato	MG735130	99%	AY836148
48	52	60	M	Ninh Binh	9/3/2016	Kato	MG735131	100%	AY836148
49	53	54	F	Son La	10/8/2016	Kato	MG735132	99%	AY836148
50	35	51	F	Son La	22/8/2016	Kato	MG735141	99%	AY836148

51	10	63	F	Nam Dinh	16/5/15	Kato	MG735127	97%	AY836148
52	12	63	F	Nam Dinh	16/5/15	Kato	MG735128	97%	AY836148
53	38	42	M	Vinh Phuc	15/7/15/	Kato	MG735135	97%	AY836148
54	137	39	F	Ha Tinh	7/5/2016	Kato	MG735136	97%	AY836148
55	152	63	F	Ha Noi	29/5/2016	Kato	MG735137	96%	AY836148
56	173	72	F	Ha Noi	27/6/2016	Kato	MG735138	97%	AY836148
57	184	33	M	Hai Phong	20/4/15	Kato	MG735139	97%	AY836148
58	12	37	F	Vinh Phuc	18/5/2016	Kato	MG735140	97%	AY836148
59	36	67	F	Ha Noi	9/9/2016	Kato	MG735142	97%	AY836148
60	72	69	M	Nam Dinh	14/6/2016	Kato	MG735133	100%	GQ332763
61	84	16	F	Cao Bang	3/10/2016	Kato	MG735134	99%	GQ332763
62	28	43	F	Ha Noi	26/6/15	Kato	MG872769	98%	AY836148
63	32	59	M	Ninh Binh	8/7/2015	Kato	MG872770	100%	AY836148
64	57	31	F	Ha Noi	26/03/2016	Kato	MG872767	99%	GQ332763
65	107	44	F	Hai Phong	4/7/2017	Kato	MG920489	97%	AY836148
